# Impact of intraepithelial capillary loops and atypical vessels in confocal laser endomicroscopy for the diagnosis of laryngeal and hypopharyngeal squamous cell carcinoma

**DOI:** 10.1007/s00405-021-06954-8

**Published:** 2021-06-29

**Authors:** Matti Sievert, Markus Eckstein, Konstantinos Mantsopoulos, Sarina K. Mueller, Florian Stelzle, Marc Aubreville, Nicolai Oetter, Andreas Maier, Heinrich Iro, Miguel Goncalves

**Affiliations:** 1 Department of Otorhinolaryngology, Head and Neck Surgery, Friedrich-Alexander-Universität Erlangen–Nürnberg, University Hospital, Waldstrasse 1, 91054 Erlangen, Germany; 2 Institute of Pathology, Friedrich-Alexander-Universität Erlangen–Nürnberg, University Hospital, Erlangen, Germany; 3 Department of Maxillofacial Surgery, Friedrich-Alexander-Universität Erlangen–Nürnberg, University Hospital, Erlangen, Germany; 4 Institute of Image Understanding and Medical Application of Artificial Intelligence, Technische Hochschule, Ingolstadt, Germany; 5 Pattern Recognition Lab, Computer Science, Friedrich-Alexander-Universität Erlangen–Nürnberg, Erlangen, Germany

**Keywords:** Confocal laser endomicroscopy, Head and neck squamous cell carcinoma, Capillary loops, Malignant vascularization, Head and neck malignancies

## Abstract

**Purpose:**

Confocal laser endomicroscopy (CLE) allows surface imaging of the laryngeal and pharyngeal mucosa in vivo at a thousand-fold magnification. This study aims to compare irregular blood vessels and intraepithelial capillary loops in healthy mucosa and squamous cell carcinoma (SCC) via CLE.

**Materials and methods:**

We included ten patients with confirmed SCC and planned total laryngectomy in this study between March 2020 and February 2021. CLE images of these patients were collected and compared with the corresponding histology in hematoxylin and eosin staining. We analyzed the characteristic endomicroscopic patterns of blood vessels and intraepithelial capillary loops for the diagnosis of SCC.

**Results:**

In a total of 54 sequences, we identified 243 blood vessels which were analyzed regarding structure, diameter, and Fluorescein leakage, confirming that irregular, corkscrew-like vessels (24.4% vs. 1.3%; *P* < .001), dilated intraepithelial capillary loops (90.8% vs. 28.7%; *P* < .001), and increased capillary leakage (40.7% vs. 2.5%; *P* < .001), are significantly more frequently detected in SCC compared to the healthy epithelium. We defined a vessel diameter of 30 μm in capillary loops as a cut-off value, obtaining a sensitivity, specificity, PPV, and NPV and accuracy of 90.6%, 71.3%, 57.4%, 94.7%, and 77.1%, respectively, for the detection of malignancy based solely on capillary architecture.

**Conclusion:**

Capillaries within malignant lesions are fundamentally different from those in healthy mucosa regions. The capillary architecture is a significant feature aiding the identification of malignant mucosa areas during in-vivo, real-time CLE examination.

## Introduction

Squamous cell carcinoma (SCC) is considered responsible for over 90% of all cancers of the pharynx and nearly 100% of the larynx malignancies [1]. Currently, the gold standard of diagnosis is a tissue biopsy followed by a histopathological assessment. Furthermore, the biopsy and the resected tumor tissue provide information about the surgical margins: When so-called margin specimens are taken, healthy tissue is biopsied to demonstrate a resection in sano in the intraoperative frozen section. Due to the invasiveness and the limited sample size in some cases, the definition of clear resection margins is not always easy. It may lead to an increased resection defect on one side and non in sano resection on the other side [2].

In recent years, probe-based Confocal Laser Endomicroscopy (CLE) has been intensively studied in gastroenterology, pneumology, and neurosurgery. This method has been successfully applied for visual inspection of suspicious mucosal lesions, magnifying power up to 1000 times using Fluorescein to outline the intercellular spaces and visualization of blood vessels [3]. Due to this property, CLE is said to provide “real-time” optical biopsies [4]. In pathological tumor angiogenesis, there is permanent overstimulation and interruption of the regular angiogenesis cascades, which causes excessive neovascularization and the formation of disorganized, tortuous, and leaky vascular convolutions. These atypical blood vessels are often insufficient in supplying central tumor tissue portions, contributing to tumor necrosis [5, 6]. As one of the essential criteria for differential diagnosis of benign versus malignant lesions in conventional histopathology, nuclear morphology cannot be adequately visualized with Fluorescein-CLE, the only drug-device combination with regulatory FDA approval. Fluorescein is distributed through the intercellular spaces and cytoplasmic components without staining the cell nuclei directly [4, 7]. Other contrast agents such as topic Acriflavine [8] or antibody-based [9] are either carcinogenic or still in an experimental stage.

However, CLE can resolve tissue architecture alterations, cell crowding, cell size, and, notably, blood vessel architecture properly [10, 11]. This study aimed to assess the impact of intraepithelial capillary loops (ICL) and atypical vessels for the diagnosis of laryngeal squamous cell carcinoma via CLE.

## Materials and methods

### Study design

This prospective pilot study was conducted at a tertiary hospital and academic cancer center (Department of Otorhinolaryngology, Head and Neck Surgery, Friedrich Alexander University of Erlangen-Nuremberg, Erlangen, Germany). The study was approved by the local institutional ethics committee (approval number 60_14 B) and carried out following the Declaration of Helsinki. We obtained written informed consent from all study participants.

### Eligibility criteria

A total of ten patients with confirmed SCC and planed total laryngectomy were included in this study between March 2020 and February 2021. Exclusion criteria were a prior treatment of any head and neck cancer, distant metastasis, radiotherapy in the head and neck area, pregnancy, thyroid dysfunction, severe kidney failure, and allergy to Fluorescein.

### Confocal laser endomicroscopy system and data acquisition

We performed intraoperative imaging using a GastroFlex probe and a 488 nm Cellvizio^™^ laser scanning system (Mauna Technologies, Paris, France). The probe has a diameter of 2.6 mm, a penetration depth of 55–65 µm, a field of view of 240 µm, and a resolution of 1 µm. We used 5 ml, Fluorescein Alcon, 10% (Alcon PHARMA, Freiburg, Germany) as an optical imaging agent. Surgery began with elevation of the apron flap, following mobilization of the larynx. The second step was to perform a pharyngotomy with cold instruments to avoid thermocoagulation damage to the mucosa. The installation of the CLE probe followed the exposure of the tumor. Subsequently, 2.5 ml Fluorescein Alcon 10% was injected intravenously. After around 8–10 min of examination, additional 2.5 ml were administrated to increase the quality of imaging. We collected images of the marginal tumor region and the incision margin in the hypopharyngeal mucosa with the CLE probe. The recorded areas were marked with a suture, or a separate biopsy was performed at the precise location of the image acquisition, below the 2.8 mm CLE probe. In this way, CLE imaging could be correlated with the gold standard of histopathology, as the same mucosal region was assessed. The histopathological assessment followed a standard protocol with hematoxylin and eosin (H&E) staining. After completing the CLE examination, we performed the tumor resection with a macroscopic safety margin of 1 cm. Our and international treatment standards were not altered or influenced in any way by the application of CLE.

### Evaluation of the vessel architecture in CLE

Based on correlation with histology, irregularly arranged, atypical vessels and altered ICL in terms of caliber, number, and shape, were distinguishing features between cancerous and normal tissue. For data processing, we analyzed the sequences using Cellvizio Viewer software 1.6.2. Each frame was examined individually by the investigators for the presence of blood vessels. We only analyzed blood vessels that were fully imaged and recorded in acceptable quality, without noise or motion artifacts. Atypical blood vessels (with a corkscrew-like shape or tangentially running vessels) were scored in a dichotomous fashion (present/absent). We measured each ICL identified in the sequences in acceptable quality in two diameters. We defined the diameter of the ICL as the longest dimension of the ICL with the largest cross sectional area. Fluorescein leakage was recorded, and the leakage zone (distance from the capillary edge and the maximum extension of the Fluorescein spot) was measured.

### Statistical analysis

The two-tailed *t* test for independent samples was applied to all continuous, normally distributed variables. For the categorical variables, we used the Chi-squared test. We performed a receiver operating characteristic (ROC) curve to determine the optimal cut-off value to maximize the sensitivity, specificity, and accuracy and the positive predictive (PPV) and negative predictive value (NPV). Diagnostic parameters are presented in percentages and the 95% confidence interval (95%CI). A *P* value of less than *P* < 0.05 was considered statistically significant. We performed statistical analysis using SPSS version 22.0 (IBM SPSS Statistics for Windows, Version 22.0. Armonk, NY, USA).

## Results

### Patient cohort

In total, we included ten patients (nine men and one woman with an average age of 50.1 ± 11.2 years) with histologically confirmed laryngeal/hypopharyngeal SCC in this study. All patients underwent radical tumor resection (total laryngectomy with or without partial pharyngectomy) as part of surgical treatment. In six patients, we performed reconstruction using a free microvascular flap. We achieved complete tumor resection with free margins in each case. The patient cohort is presented in Table 1.

**Table 1:** Patient cohort

Case	Age	Sex	Localization	TNM	Grade	Procedure	Sequences	Frames
1	58	Male	HP	T4aN0M0	G3	LE with free flap reconstruction	6	1670
2	80	Male	L	T3N0M0	G3	LE	6	1681
3	67	Male	HP	T3N3bM0	G3	LE with free flap reconstruction	7	5639
4	53	Female	L	T4aN2bN0	G2	LE	5	1817
5	66	Male	HP	T3N0M0	G3	LE with free flap reconstruction	2	172
6	71	Male	HP	T4aN2bM0	G3	LE with free flap reconstruction	3	1468
7	56	Male	HP	T4aN3bM0	G3	LE with free flap reconstruction	6	2204
8	86	Male	L	T4aN0M0	G2	LE	5	2191
9	61	Male	L	T3N0M0	G3	LE	8	3311
10	53	Male	HP	T2N2bM0	G3	LE with free flap reconstruction	6	2891

*HP* hypopharynx, *L* larynx, *LE* laryngectomy

A total of 23061 images (on average 2304 ± 1370 images in each patient) from 54 sequences (on average 5 ± 2 sequences in each patient) were analyzed concerning the presence of atypical vessels and the number and diameter of ICLs, as well as the occurrence and extent of capillary leakage. We correlated each sequence with a specimen as a standard of reference. Thus we histologically confirmed SCC in 29 sequences (on average 426 ± 291 images) and benign squamous epithelium in 25 sequences (on average 428 ± 241 images; *P* = 0.977). All benign mucosal specimens were free of dysplasia or carcinoma in situ.

### Atypical vessels and ICLs in benign and malignant mucosa

A total of 243 vessels were identified with adequate quality for data analyses. Considering H and E staining as a reference standard, 157 blood vessels (64.6%) correlated with benign, and 86 blood vessels (35.4%) correlated with malignant mucosa. We observed a significantly higher proportion of irregular arranged, atypical blood vessels in SCC areas (21/86; 24.4%) compared with healthy mucosa (2/157; 1.3%; *P* < 0.001). Atypical blood vessels show a horizontal course in the upper epithelial layers, caliber changes, and a “corkscrew-like” shape. Considering the number of ICLs (*n* = 220; in a total of 23061 images), we could assign 155 ICLs (70.5%) to benign mucosa (10705 images) and 65 ICLs (29.5%) to malignant epithelium (12356 images; *P* < 0.001). We observed capillary leakage in 49 blood vessels (57.0%) of malignant mucosa and 21 blood vessels (13.4%) of benign mucosa (*P* < 0.001).

### Receiver operating characteristic curve analysis of the vessel diameter and capillary leakage

**Fig. 1:**
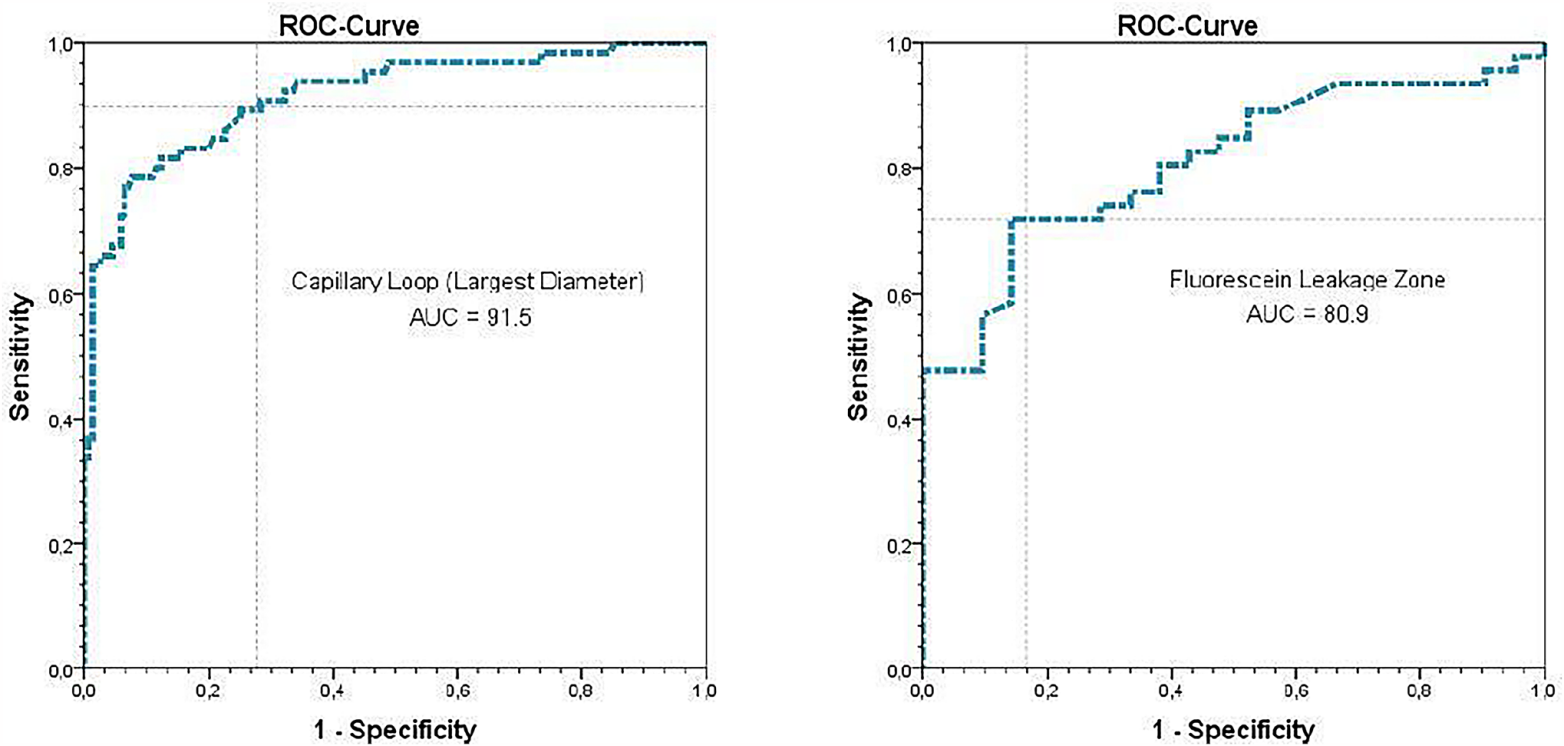
Receiver operating characteristic curves regarding the intraepithelial capillary loop diameter and the Fluorescein leakage zone. We determined a cut-off value of 30 and 40 μm to achieve the highest sensitivity and specificity, respectively

Considering the largest vessel diameter in ICLs, we detected a significant difference in the benign and malignant epithelium, measuring 25.1 μm (SD = 9.9) and 54.9 μm (SD = 22.1), respectively (*P* < 0.001). Subsequently, a ROC curve was plotted for the largest vessel diameter in SCC and healthy tissue to determine the cut-off value with the highest sensitivity and specificity (Fig. 1). We calculated an area under the curve (AUC) of 91.5 (95%CI 87.1–95.9). The optimal cut-off value between the two groups was defined as 30 μm. Applying this decision criteria, we calculated sensitivity, specificity, PPV, and NPV and accuracy of 90.6%, 71.3%, 57.4%, 94.7%, and 77.1%, respectively (Table 2), with a significantly higher proportion of dilated ICLs in malignant tissue (90.8% vs. 28.7%; *P* < 0.001). Concerning the maximum leakage zone, we were able to calculate an AUC of 80.9. The leakage zone measured on average 4.1 μm (SD = 11.5) and 33.5 μm (SD = 39.0) for benign mucosa and SCC, respectively (*P* < 0.001). The optimal cut-off value was defined as 40 μm and achieved a sensitivity, specificity, PPV, NPV, and accuracy of 84.6%, 71.4%, 89.2%, 62.5%, and 81.15%, respectively (Table 2), with a significantly higher Fluorescein leakage ≥ 40 μm in malignant tissue (40.7% vs. 2.5%; *P* < 0.001). Considering an ICL diameter of ≥ 30 μm and a leakage of ≥ 40 μm, we calculate sensitivity, specificity, PPV, NPV, and accuracy of 44.6%, 98.1%, 90.6%, 80.8%, and 82.3%, respectively (Table 2).

**Table 2:** Comparison of endomicroscopic findings in malignant and benign mucosa

CLE parameter	SCC (*n* = 86)	Benign Mucosa (*n* = 157)	*P* value	Sensitivity % (95%CI)	Specificity % (95%CI)	PPV % (95%CI)	NPV % (95%CI)
Irregular/atypical vessels; *n* (%)	21 (24.4)	2 (1.3)	< 0.001	–	–	–	–
ICL count; *n* (%)	65 (75.6)	155 (98.7)	< 0.001	–	–	–	–
ICL diameter (μm)^a^	54.9 ± 22.1	25.1 ± 9.9	< 0.001	90.6 (80.7–96.5)^a^	71.3 (63.4–78.4)^a^	57.4 (50.9–63.7)^a^	94.7 (89.2–97.5)^a^
Capillary leakage; *n* (%)	49 (57.0)	21 (13.4)	< 0.001	–	–	–	–
Capillary leakage zone (μm)^b^	33.5 ± 39.0	4.1 ± 11.5	< 0.001	84.6 (69.5–94.1)^b^	71.4 (41.9–91.6)^b^	89.2 (78.1–95.0)^b^	62.5 (42.6–78.9)^b^
ICL diameter ≥ 30 μm^a^ and capillary leakage ≥ 40 μm^b^; *n* (%)	29 (33.7)	3 (1.9)	< 0.001	44.6 (32.3–57.5)^ab^	98.1 (94.4–99.6)^ab^	90.6 (75.3–96.8)^ab^	80.8 (77.2–84.0)^ab^

*CLE* confocal laser endomicroscopy, *SCC* squamous cell carcinoma, *ICL* intraepithelial capillary loops

^a^cut-off value ≥ 30 μm

^b^cut-off value ≥ 40 μm

## Discussion

**Fig. 2:**
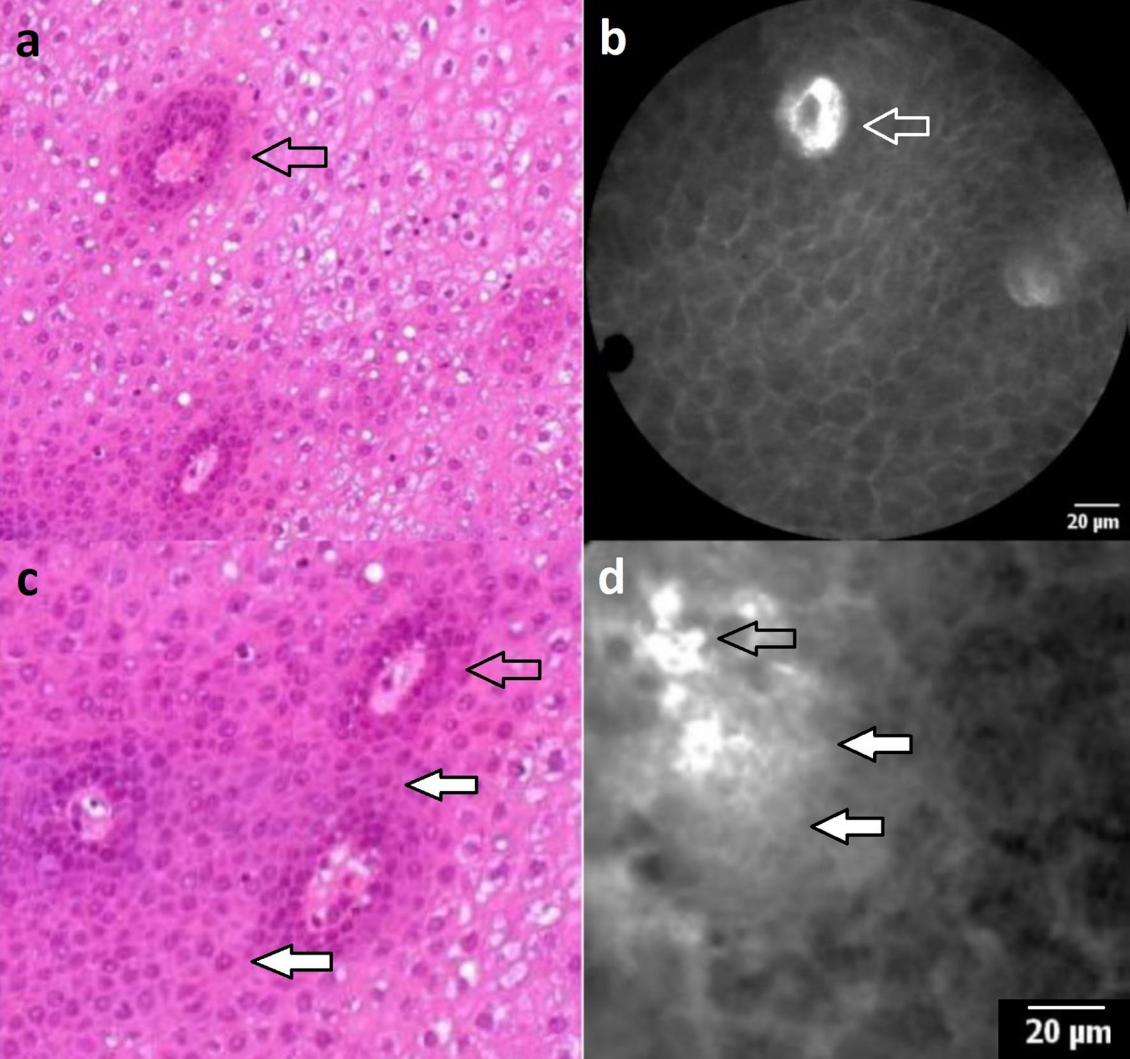
**a**, **c** Hematoxylin and Eosin (HE) stained section from squamous epithelium of the hypopharyngeal space. The epithelium is cut tangentially to reflect HE morphology in the same dimension as CLE **b**, **d**. Epithelium shows slight reactive changes but no dysplasia. The transparent arrows indicate capillaries. The white arrows mark the inflammatory infiltrate with abundant lymphocytes **d** and a fluorescent dye leakage **d**. Thus, the capillary loops each measure less than 30 μm in their longest diameter, and therefore, are not considered suspicious

In this study, we evaluate the capability of assessing the superficial blood vessels in SCC and normal mucosa of the larynx and hypopharynx by CLE and attest criteria to differentiate between benign and malignant epithelium. Based on the present data, we can confirm that irregular vessels, with an atypical and horizontal course in the upper epithelial layers and a corkscrew-like shape, are significantly associated with malignant mucosa (*P* < 0.001). Since the regular vascular course in healthy squamous mucosa is vertical, ICLs inherently indicate non-suspicious mucosal architecture (Fig. 2a). We registered that the sole number of ICLs is significantly higher in healthy epithelial areas than the tumor's marginal areas (*P* < 0.001). However, deformed capillaries with an expanded diameter (54.9 μm vs. 25.1 μm; *P* < 0.001) and increased leakage of a fluorescent dye (33.5 μm vs. 4.1 μm; *P* < 0.001) in SCC are noticeable.

**Fig. 3:**
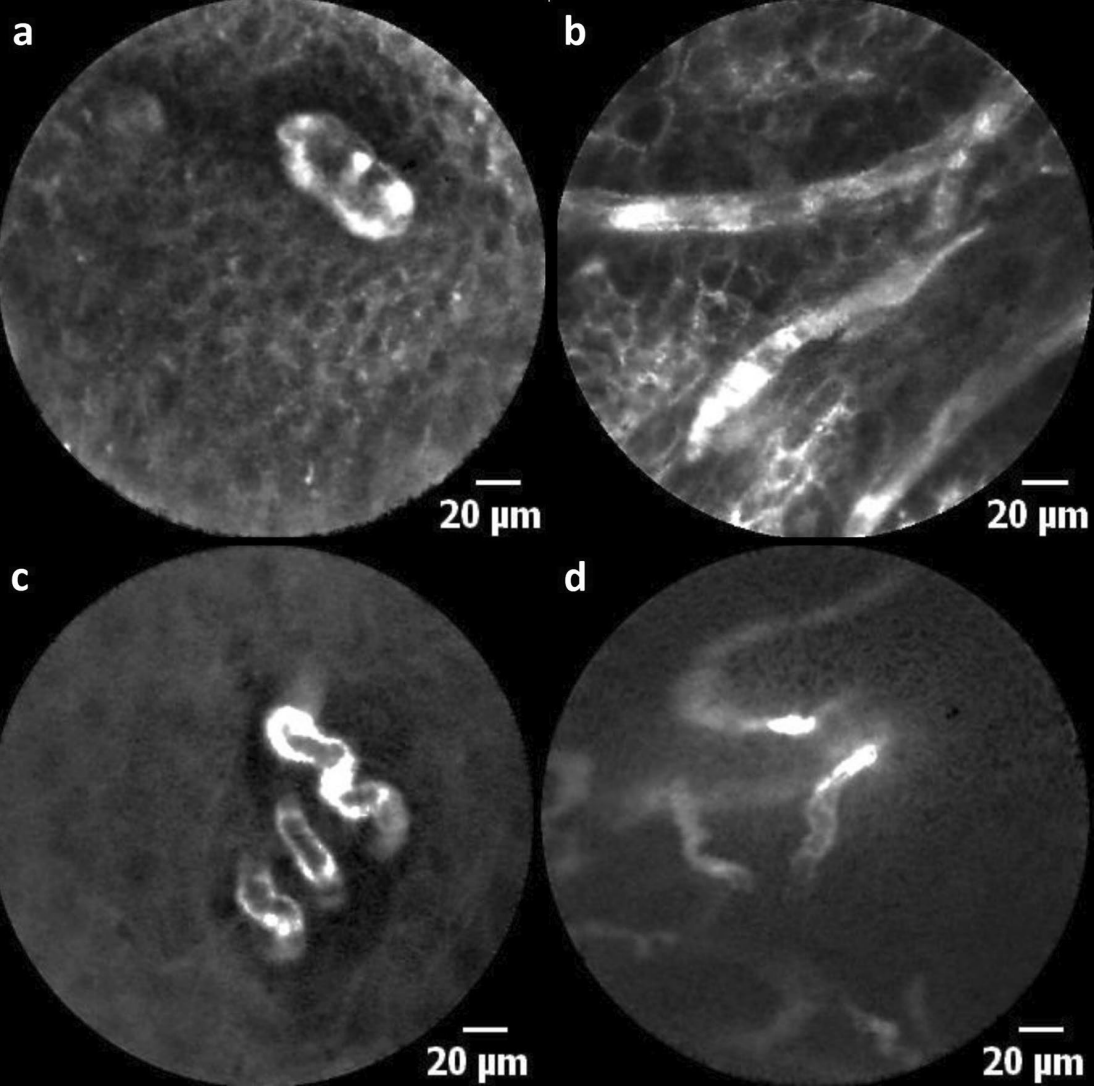
Atypical blood vessels: **a** dilated, oval-shaped intraepithelial capillary loop and **b** enlarged horizontal running blood vessels in the upper epithelial layers at the tumor border. **c** Corkscrew-like vessel shapes and **d** diffusely branched irregular blood vessels in the tumor center

ICLs have been described as a classic characteristic of the mucosa in the esophageal region. Intrapapillary capillaries arise from arborescent vessel’s outlets into the epithelial papillae and form individual loops called ICLs [12]. Their occurrence is not limited to the esophageal mucosa and is typically found also in the laryngeal, hypo-, and oropharyngeal regions and the oral cavity [10, 13–15]. Healthy epithelium ICLs show a round shape in the orthogonal plane view without leakage of the fluorescent dye (Fig. 2a, b). In contrast, ICLs of altered neoplastic mucosa are often blurred and show an oval, dysmorphic shape (Fig. 3a). We can determine a cut-off value of 30 μm to distinguish between normal and malignant epithelial clusters. Our results are consistent with studies in patients with esophageal cancer, confirming a cut-off value of 26–30 μm for SCC diagnosis [16, 17].

Although nuclear morphology, one of the essential criteria for differential diagnosis of benign versus malignant lesions in conventional histopathology, cannot be adequately resolved by CLE, it can determine alterations of tissue architecture, cell crowding, cell size, and blood vessel architecture properly. While sole assessment of capillary permeability as a criterion for malignancy is not recommended due to its frequent occurrence in inflammatory tissue (Fig. 2c, d), especially the combination of atypical vessel architecture and increased capillary permeability seems to allow accurate prediction of malignant versus benign lesions, which is supported by data from gastroenterological studies [18, 19]. Considering an ICL diameter of ≥ 30 μm and a capillary leakage of ≥ 40 μm, we demonstrated high specificity of 98% for SCC detection with a low sensitivity of 44.6%, however. Therefore, we recommend emphasizing the ICL diameter in diagnosing SCC, reaching a sensitivity of 90.6%. However, when determining malignancy based on sole vessel morphology and permeability, caution must be exercised. We could detect ICLs in benign as well as malignant mucosal areas. Therefore, the presence of ICLs is not a specific sign of benignity. The same applies to the presence of vessel leakage. Although leakage is commonly seen in SCC, its absence is also not a specific criterion of healthy tissue. It is crucial to consider that leakage of fluorescent dye surrounding the blood vessel may also occur due to an inflammatory exudate (Fig. 2d) and is not further differentiated by CLE. Although information regarding blood vessels can contribute to malignancy assessment, it may not be sufficient as a stand-alone criterium. Present data provided by this manuscript helps define vascular structures better and, thus, in combination with other cell and tissue features, eventually improves the diagnostic metrics for dichotomous classification healthy / SCC. A more objective approach is to consider the entire image morphology, considering cell shapes, cell sizes, and the overall context of the cell cluster.

Another limiting aspect of CLE is the planar imaging of the epithelial surface in a perpendicular orientation to the usual histologic section (i.e., parallel to the surface, at a depth defined by the probe) [20]. The tissue penetration depth ranges from 60 to 350 µm, depending on the specific system [21]. Therefore, CLE is limited to exclusively delivering tangential images of analyzed tissue areas, thus not allowing to differentiate epithelial lesions based on absence or presence of invasiveness leading to a significantly complicated differential diagnosis of invasive carcinoma versus in situ carcinoma.

However, we can predict malignant lesions with high sensitivity and specificity using cell’s shape and configuration. Previous studies demonstrated the feasibility of intraoperative definition of the incision margin with acceptable sensitivity and specificity in laryngeal and oropharyngeal squamous cell carcinoma [13, 14, 22]. In a comprehensive analysis of CLE images from the oral cavity, Oetter et al. described a scoring system to facilitate classification of the malignancy of mucosal lesion’s denitrification parameters include the morphological criteria “homogeneity,” “intercellular gaps,” “cell morphology,” "Fluorescein leakage,” and “vessel morphology,” obtaining a sensitivity of 97.3% and a specificity of 88.1%. The authors made no definite statement regarding the vessel caliber and the extent of vessel leakage [10]. All raters were asked to dichotomously evaluate atypical vessels' presence and the presence of leakage, respectively. We validated these evaluation criteria on mucosal scans of the oropharynx and hypopharynx or larynx in a previously unpublished study and obtained comparable results with a sensitivity and specificity of 81.3% and 85.5%, respectively, which essentially attest to the transferability of the criteria from the oral mucosa to pharyngeal and laryngeal, but also suggest some fine-tuning of these criteria may be required. The differences regarding the sensitivity can be attributed to a higher inhomogeneity of the different anatomical regions in our study cohort. The present data indicate that a more precise consideration of a single morphologic criterion may have comparable diagnostic significance. Therefore, the detailed observation and measurements of distinct morphological characteristics is a worthwhile attempt.

Belykh et al. examined the presence of thrombosis, flow velocity changes, and agglutination in gliomas during neurosurgery, demonstrating the feasibility and potential of examining capillaries by CLE. It was demonstrated that lymphatic channels could also be differentiated from blood vessels [23]. Further research is needed to evaluate the transferability of these results in the head and neck region. Investigating these parameters to classify vascular and lymphatic mucosal structures could refine and redefine present classification systems, which only broadly regard vessel morphology, as stated above [10]. A precise classification system is paramount to an eventual narrowing of acceptable safe margins, which at this point cannot be recommended. Computer-aided detection of vascular structures may be a more advanced approach. Mahé et al. developed an image processing method that enhances visualization of moving structures such as blood cells in capillaries, providing a dynamic representation of the imaged tissue [24]. Dittberner et al. demonstrated that classification of CLE scans into normal and malignant altered tissue is possible based on an estimate of mean cell sizes [25]. Aubreville et al. were able to determine mucosal lesions' malignancy based on deep learning algorithms using CLE scans of different regions of the upper aerodigestive tract [26]. However, since all investigations to date have been performed on a small number of hand-selected prototypical images, the results should not be generalized, and broad day-to-day application based on automatic algorithms is not yet viable outside experimental studies for the head and neck region. The inclusion and labeling of information regarding vascular structures validated by this work, such as a cut-off value to healthy capillaries of 30 μm, could potentially improve the definition of algorithms to classify the images provided.

In CLE, visualization and analysis of vascular architecture are pivotal for distinguishing malignant and benign squamous mucosa. The vascular images were recorded in the tumor's marginal area, close to the macroscopically inconspicuous mucosa transition, determining that only rare blood vessels are detectable in the tumor center by using CLE. The central tumor necrosis zone may be causative for this observation. Nevertheless, the marginal area of the tumor is of particular importance for surgical therapy. With increasing experience and refinement of diagnostic criteria, CLE offers the potential to define resection margins more accurately. This technique can be beneficial for tumor resection and therapy planning, as the expected extent of resection can be more precisely delineated.

## Conclusion

The precise analysis of vascular structures is a promising approach to improving CLE’s diagnostic value to differentiate malignant squamous epithelial lesions and benign mucosa. In addition to blood vessel morphology, the caliber and vascular leakage should be an integral part of the evaluation criteria for CLE images. In the present study, increased diameter of ICLs, tortuous and irregular-shaped blood vessels were valuable features in distinguishing SCC patients.
